# Analysis of epigenetic biomarkers for diagnosis and assessment of severity in rheumatoid arthritis: a cross-sectional study

**DOI:** 10.1186/s13075-025-03628-2

**Published:** 2025-08-08

**Authors:** Arkaitz Mucientes, Gracia María Martín Núñez, Natalia Mena-Vázquez, Jose Manuel Lisbona-Montañez, Sara Manrique-Arija, Andrés González-Jiménez, Patricia Ruiz-Limón, Aimara Garcia-Studer, Fernando Ortiz-Márquez, Laura Cano-García, Antonio Fernández-Nebro

**Affiliations:** 1 Instituto de Investigación Biomédica de Málaga y Plataforma en Nanomedicina-IBIMA Plataforma, BIONAND, Málaga, 29590 Spain; 2 UGC de Reumatología, Hospital Regional Universitario de Málaga, Málaga, 29009 Spain; 3 Departamento de Medicina, Universidad de Málaga, Málaga, 29010 Spain; 4 Bioinformatic platform, Instituto de Investigación Biomédica de Málaga y Plataforma en Nanomedicina-IBIMA Plataforma BIONAND, Málaga, 29590 Spain; 5 Unidad de Gestión Clínica de Endocrinología y Nutrición, Hospital Clínico Virgen de la Victoria, Málaga, 29010 Spain; 6 CIBER Fisiopatología de la Obesidad y Nutrición (CIBEROBN), Instituto de Salud Carlos III, Madrid, 28029 Spain

**Keywords:** Biomarkers, Epigenetic, Inflammation, Methylation, Rheumatoid arthritis

## Abstract

**Background:**

Rheumatoid arthritis (RA) is an autoimmune disease influenced by genetic, environmental, and epigenetic factors. Epigenetic modifications, particularly DNA methylation, in immune-related genes may impact inflammation and immune responses. This study aims to analyze methylation patterns in RA patients and controls to identify diagnostic and prognostic epigenetic biomarkers.

**Methods:**

A cross-sectional study of a prospective cohort comprising 32 patients (16 with severe RA, 16 with nonsevere RA) and 32 healthy controls (discovery cohort) was performed. Severity was defined as a cumulative 28-joint Disease Activity Score with erythrocyte sedimentation rate (DAS28-ESR) ≥ 3.2, positive rheumatoid factor (RF) and anti–citrullinated peptide antibody (ACPA) values, and a high *Collinsella aerofaciens* count (OTU ≥ 0.15). Whole genome methylation analysis was performed using the Infinium Methylation EPIC BeadChip kit. Subsequently, validation by pyrosequencing (PyroMark Q48) was performed for the differentially methylated positions (DMPs) selected both in the discovery cohort and in the remainder of the inception cohort (78 patients and 78 controls).

**Results:**

More than half of the participants were women (≥ 75%), and the mean age was 56 years. At whole genome level, an epigenetic signature was associated with both RA and severity of RA. Pyrosequencing confirmed that methylation levels at CpG sites in *TBC1D22A*, *PRHOXNB*, *ALLC*, and *PRG2* genes were associated with RA or severity of RA. The novel association between hypermethylation in *TBC1D22A* and RA was subsequently confirmed in an independent cohort.

**Conclusions:**

Our results indicate that the level of DNA methylation in validated DMPs is associated with RA. Thus, these methylation levels are potential biomarkers for the diagnosis, prognosis and severity of RA.

**Supplementary Information:**

The online version contains supplementary material available at 10.1186/s13075-025-03628-2.

## Background

Rheumatoid arthritis (RA) is an immune-mediated inflammatory disease characterized by chronic synovitis. If left untreated, it causes progressive and irreversible joint destruction and functional disability. It has been estimated that RA affects 0.5-1% of the adult population worldwide [1]. The precise etiology of RA remains the subject of ongoing research, and while not fully understood, it is widely acknowledged that RA results from a multifaceted interplay of various factors. Genetic predisposition plays a crucial role, as certain gene variants are associated with increased susceptibility to the disease. However, genetics alone cannot account for all aspects of the development of RA, and it is believed that environmental, hormonal, and immunopathological factors also contribute significantly to pathogenesis in a multistep process [2, 3].

Since epigenetic modifications could be the link between genetic and environmental factors related to the onset and course of RA [4], several works have tried to shed light on the role of epigenetics in the onset of RA. In this sense, it has been proven that various methylation sites present in synovial fibroblasts and peripheral blood mononuclear cells at gene promoter loci (e.g., CXCL12, DR3, IL-6, IL-10, and IL-1R2) may affect the immune response, inflammation, and leukocyte recruitment [5–7]. Thus, it has been reported that hypomethylation of death factor 3 in synovial RA causes resistance to apoptosis and significant changes in methylation patterns of both B and T cells from RA patients [8].

A study on the DNA methylation profile of the interleukin 6 (IL-6) gene promoter in patients with RA, patients with chronic periodontitis, and healthy controls found that the methylation levels of a specific CpG site were significantly lower in RA and chronic periodontitis patients than in controls, leading to increased levels of serum IL-6 [9]. These findings suggest that epigenetic modifications play a significant role in the pathogenesis of RA, impacting the immune response, inflammation, and the behavior of specific immune cells. Furthermore, the intestinal microbiota is altered in patients with RA [10]. Our group has reported relevant data on microbiota abnormalities associated with the degree of disease activity, concluding that the genus *Collinsella* seems to play an important role in cumulative inflammatory burden in patients with established RA [3]. Understanding this connection could reveal an association between specific environmental and clinical factors, the patient’s epigenetic profile, and changes in the intestinal microbiota [11]. Therefore, the objective of this study was to analyze methylation patterns in patients with RA and healthy controls and identify epigenetic markers that could predict the severity of the disease.

## Methods

### Design and scope of the study

The study population comprised 110 RA patients and 110 healthy controls selected from an inception cohort recruited between 2007 and 2011 and followed up prospectively in the Rheumatology Department of Hospital Universitario Regional in Málaga, Spain. The details of this cohort have been published elsewhere [3, 12]. The study was approved by the local medical ethics committee, and all participants provided their written informed consent before enrolling (Project identification code 4/2016, P19).

### Participants

The inclusion criteria were as follows: age ≥ 18 years; RA diagnosed according to the 2010 criteria of the American College of Rheumatology/European League Against Rheumatism [13]; and having been diagnosed and treated within 12 months of the onset of RA. Patients with rheumatic disease other than RA, except for secondary Sjögren’s syndrome, were excluded. The patients included were distributed into two cohorts, one for discovery and the other for technical validation.

#### Discovery cohort

This cohort comprised 32 patients selected from the inception cohort, 16 in each group, with an extreme phenotype according to the presence/absence of severity factors, and 32 healthy controls, matched by sex and age for array analysis and technical validation by pyrosequencing of selected CpGs. The extreme phenotype with severity factors (16 patients) was defined as a cumulative inflammatory burden of moderate-high activity according to the 28-joint Disease Activity Score with erythrocyte sedimentation rate (DAS28-ESR ≥ 3.2), positivity for both rheumatoid factor (RF) and anti–citrullinated peptide antibody (ACPA), and a high abundance of Collinsella aerofaciens (OTUs ≥ 0.10). The extreme phenotype without severity factors (16 patients) was defined as a cumulative inflammatory burden of remission–low activity (DAS28-ESR < 3.2) in patients matched by sex and age with those of the extreme phenotype group [3, 12].

#### Validation cohort

The remaining patients from the inception cohort (78 patients with RA) and 78 matched healthy controls were selected for the validation study by pyrosequencing of the selected CpGs. Patients with the severe and nonsevere phenotype were stratified as DAS28-ESR low and DAS28-ESR moderate/high.

### Laboratory and clinical variables

All participants were seen at the clinic between 2018 and 2020, and all data were entered into a database designed according to the protocol. Demographic, clinical, laboratory, and treatment-related data were recorded by a rheumatologist. Demographic variables included age (in years), sex, and race. We also collected conventional cardiovascular risk–related variables, as follows: smoking (active, ex-smoker, never), obesity (body mass index > 30) [14], arterial hypertension ≥ 140/90 mmHg or current antihypertensive medication [15], diabetes diagnosed according to the criteria of the American Diabetes Association, and a personal history of cardiovascular disease [16].

The characteristics of RA patients recorded were as follows: date of disease onset, disease duration was calculated as the time from diagnosis to the sample collection date and a series of analytical data, namely, RF (reference value 20 U/ml; high titers > 60 U/ml), ACPA (reference value 10 U/ml, high values > 340 U/ml), C-reactive protein (mg/dl), and ESR (mm/h). The cumulative inflammatory burden in each patient was assessed using the DAS28-ESR (range 0-9.4) recorded at each visit since the start of the cohort [3, 17]. According to the DAS28-ESR, disease activity was defined as high-moderate (≥ 3.2) and low-remission (< 3.2). We also recorded severity-related variables such as the presence of radiological erosions and physical function (evaluated based on the mean Health Assessment Questionnaire [HAQ] score throughout the course of the disease) [18], as well as the levels of *Collinsella aerofaciens* in the intestinal microbiota [3]. Information on treatments with conventional synthetic DMARDs (csDMARDs), biological DMARDs (bDMARDs), and corticosteroids at the time of sample collection was recorded.

### Genome‑wide dna methylation assay

Genomic DNA was extracted from 200 µl of buffy coat using the DNeasy^®^ Blood & Tissue Kit (QIAGEN). DNA purity and quantity were evaluated using NanoDrop (Thermo Fisher, CA, USA) and Qubit (Life Technologies, CA, USA) devices, respectively. For bisulfite conversion, 500 ng of DNA was treated using the EpiTect Bisulfite (QIAGEN) DNA methylation kit following the manufacturer’s recommendations. All bisulfite-converted DNA samples were stored immediately at − 20˚C until use.

DNA methylation assays were performed using the Illumina Methylation EPIC BeadChip (EPIC) device (Illumina, CA, USA). Bisulfite-converted DNA from 32 RA patients and 32 control samples underwent HM850K hybridization according to the Illumina Infinium HD methylation protocol (https://support.illumina.com). The EPIC platform assesses the DNA methylation level of 853,307 loci around the genome at single-nucleotide resolution. Chips were scanned using an Illumina iScan SQ scanner (Illumina, CA, USA), and the fluorescence signals were interpreted using the Bioconductor packages in R (v.3.4.4). The probes were annotated from the Illumina files using UCSC version hg19 of the human reference genome. Then, the dataset was analyzed using the *minfi* package [19]. The methylation levels were obtained for each CpG site as β values on a range from 0 to 1, which is related to the methylation percentage (0–100%). One sample was discarded owing to inconsistency between the reported sex and the predicted sex based on the methylation signals in the sexual chromosomes. The normal-exponential out-of-band (Noob) preprocessing method was used for background correction. For quality control, probes with a detection *p*-value *>* 0.1 in at least 10% of samples were removed from the dataset. The probes on the EPIC have two different chemistry designs, type I and type II, which need to be normalized to make them comparable to each other [20].

#### Normalization of methylation data

Methylation signal normalization was performed using the Noob (normal-exponential out-of-band) + BMIQ (Beta MIxture Quantile dilation) approach implemented in the minfi package [21]. This strategy was chosen over Functional Normalization (FunNorm) due to the relatively small cohort size and limited batch effects, which favored an individual-sample normalization method. This approach minimizes the risk of overcorrecting true biological variation, which is particularly important in studies with reduced sample sizes. While FunNorm is effective at removing between-array variation using control probes, its more assertive adjustments may inadvertently dampen genuine epigenetic signals, potentially hindering biomarker discovery. Probes located on the X and Y chromosomes were removed. In addition, probes associated with known single-nucleotide polymorphisms and reported to be cross-hybridizing were also removed from the downstream analysis [22].

### Bioinformatic analysis

#### Differential global DNA methylation analysis

Differentially methylated positions (DMPs) were identified between groups (patients vs. controls and severe RA vs. nonsevere RA) using the Wilcox test. In addition to the phenotype, we used age and sex as confounding variables in our models. A minimum threshold of 0.10 was imposed for the difference between the mean beta values of the groups compared and *p*-values < 0.05. Therefore, only the probes with an absolute value of the difference between groups above 0.10 were taken into account for subsequent analyses. The β values were logarithmically transformed into M values, since the M values show greater homoscedasticity, generating more homogeneous and less dispersed data at the extremes than β values.

To gain insights into the underlying patterns of variations in DNA methylation, we performed principal component analysis (PCA) using the mixOmics package in R. Prior to PCA, the DNA methylation data were preprocessed to ensure comparability and remove any technical artifacts. The methylation data were normalized and log-transformed to stabilize variance across samples. A volcano plot was created with the enhanced Volcano R package using normalized microarray methylation data to visualize the global comparison between the study groups. Heatmaps were also generated using the ComplexHeatmap package in R. For the heatmap, the DNA methylation values were transformed and scaled to emphasize differences between CpGs and samples. Hierarchical clustering was performed on both rows and columns to group similar CpGs and samples together.

At the region level, the mCSEA package [23] was used to identify the differentially methylated regions (DMRs) between groups. Differential methylation was tested in promoters, gene bodies, and CpG island regions. This method makes it possible to detect DMRs even when the differential methylation of the individual CpGs is very subtle—but consistent—among the genomic regions. DMRs with *p <* 0.05 were considered significant.

#### Functional analysis

A functional enrichment analysis was performed to identify the potential mechanism that may be altered in patients compared with controls, as well as in severe RA vs. nonsevere RA. Once we obtained the DMPs with annotated genes, we ran Gene Ontology (GO) and Kyoto Encyclopedia of Genes and Genomes (KEGG) analyses. GO terms and KEGG pathways were obtained using the ClusterProfiler package in R [24]. Since the number of DMPs annotated in genes based on the criteria described above (*p*-value < 0.05 and |Δβ| >0.1) was low, DMPs annotated in genes with |Δβ| >0.05 and *p*-value > 0.05 were considered for this analysis.

### Validation of dmps by bisulfite pyrosequencing

Pyrosequencing is a quantitative method that measures DNA methylation levels (in %) for each CpG site in a specific genome region. This method was used for validation of the selected differentially methylated loci identified using the HM850k array (cg21950155, cg08161306, cg05510714, cg05073382, cg15971518, cg19052272). Pyrosequencing also included information on the neighboring CpGs, which were named with respect to the position of the CpG identified using the HM850k array (number of nucleotides with respect to the position of the CpG in the arrays-Down, downstream)/Upstream, -code CpG array). First, the CpGs selected were evaluated using pyrosequencing in the DNA sample used for HM850K hybridization (discovery cohort). CpGs with statistically significant changes in methylation levels were then validated in an independent cohort (i.e., the validation cohort).

Briefly, DNA was extracted from buffy coat using the DNeasy^®^ Blood & Tissue Kit (QIAGEN) following the manufacturer’s instructions and quantified using NanoDrop (Thermo Fisher, CA, USA). A total of 1000 ng of genomic DNA was bisulfite-treated using the EpiTect Bisulfite Kit (QIAGEN). Polymerase chain reaction assay was performed with 25 ng of bisulfite-converted DNA in a final volume of 15 µl and specific primers for each CpG site designed using PyroMark Assay Design software (version 2.0, Qiagen) (Table S1). Next, 10 µl of the amplified product was used for pyrosequencing with PyroMark Q48 Advanced CpG Reagents and the PyroMark Q48 pyrosequencer (Qiagen, Hamburg, Germany), following the manufacturer’s recommendations. CpGs with statistically significant changes (*p* < 0.05) in methylation levels were then validated in an independent cohort (i.e., the validation cohort).

### Statistical analysis

A descriptive analysis of the main demographic and clinical variables was conducted. Values were expressed as frequencies and percentages or as means (standard deviation [SD]) or medians (interquartile range [IQR]), as appropriate. Normality was assessed using the Kolmogorov-Smirnov test. Clinical and analytical characteristics were compared between patients and controls using Pearson’s χ² test or the *t* test, as appropriate. A logistic regression analysis was performed to identify CpGs associated with RA, and another was performed to identify CpGs associated with the RA severity phenotype. To evaluate the diagnostic accuracy of the selected methylation biomarkers, receiver operating characteristic (ROC) curves were constructed separately for each CpG dinucleotide. ROC curves were obtained by plotting the true-positive rate (sensitivity) on the y-axis against the false-positive rate (1-specificity) on the x-axis. By calculating the area under the curve (AUC), we measured the ability of individual CpG sites to discriminate between the sample groups analyzed. While an AUC of 1.0 reflects a perfectly accurate test, values greater than 0.7 are considered acceptable. The statistical analyses were performed using IBM SPSS Statistics for Windows, Version 28 (IBM Corp., Armonk, NY, USA).

## Results

### Baseline characteristics of the study population

Between June 2017 and September 2020, we consecutively recruited 110 patients with RA and 110 without RA. A total of 64 individuals were included in the discovery phase, namely, 32 patients with RA and the extreme severity phenotype and 32 age- and sex-matched controls. A total of 156 individuals were included in the technical validation phase, namely, 78 individuals with RA and 78 controls.

Table 1 shows the baseline characteristics of the patients and controls included in the discovery and validation phases. Consistent with these data, no significant differences were found for most of the epidemiological characteristics and comorbid conditions between the groups. The median disease duration was 84.5 months in the discovery cohort and 91.2 months in the validation cohort, with no statistically significant differences between groups (*p* = 0.570). However, compared with the control group, the patient group contained more smokers and obese individuals, and levels of *Collinsella aerofaciens* were higher. Positive antibody titers were only found among the patients. All patients in both the discovery and validation cohorts were receiving treatment with DMARDs. A total of 92.7% (102/110) of patients were taking conventional synthetic DMARDs (csDMARDs), and 38.1% (42/110) were receiving biological DMARDs (bDMARDs), mainly anti–TNF-α agents (29.1%), followed by anti–IL-6 therapies (6.4%) and rituximab (1.8%). Additionally, 18% (19/110) of patients were receiving corticosteroids. There were no statistically significant differences in the distribution of these treatments between the groups in either cohort.

**Table 1:** Baseline characteristics of patients with RA and healthy controls

	Discovery cohort			Validation cohort		
	RA *N* = 32	Controls *N* = 32	*p*-value	RA *N* = 78	Controls *N* = 78	*p*-value
*Epidemiological characteristics*						
Age, years, mean (SD)	58.1 (9.6)	57.2 (9.4)	0.690	54.9 (11.5)	54.2 (17.8)	0.771
Female sex, *n* (%)	24 (75.0)	24 (75.0)	1.000	65 (83.3)	64 (82.1)	0.560
Caucasian race, *n* (%)	32 (100.0)	32 (100.0)	1.000	76 (97.4)	77 (98.7)	0.560
BMI, kg/m^2^, mean (SD)	29.9 (5.0)	27.7 (5.2)	0.089	27.5 (4.6)	26.8 (4.2)	0.305
Smoking			0.015			0.036
Never, *n* (%)	12 (37.5)	19 (59.4)		34 (43.6)	49 (62.8)	
Ex-smoker, *n* (%)	13 (40.6)	3 (9.4)		20 (25.6)	10 (12.8)	
Active, *n* (%)	7 (21.9)	10 (31.3)		24 (30.8)	19 (24.4)	
*Comorbid conditions*						
Arterial hypertension, *n* (%)	10 (31.3)	8 (25.0)	0.578	19 (24.4)	17 (21.8)	0.704
Diabetes mellitus, *n* (%)	2 (6.3)	0 (0.0)	0.151	4 (5.1)	2 (2.6)	0.405
Dyslipidemia, *n* (%)	11 (34.4)	5 (15.6)	0.083	13 (16.7)	17 (21.8)	0.416
Obesity WHO, *n* (%)	17 (53.1)	9 (28.1)	0.042	23 (29.5)	16 (20.5)	0.339
*Clinical-laboratory characteristics*						
Time since diagnosis of RA, months, median (IQR)	84.5 (77.5-100.5)	-	-	91.2 (77.6–120.0)	-	-
Diagnostic delay, months, median (IQR)	8.3 (4.0-12.3)	-	-	8.1 (4.5–14.2)	-	-
Erosions, *n* (%)	23 (71.9)	-	-	49 (62.8)	-	-
RF > 10 U/ml, *n* (%)	29 (90.6)	0 (0.0)	< 0.001	63 (80.8)	0 (0.0)	< 0.001
ACPA > 20 U/ml, *n* (%)	23 (71.9)	0 (0.0)	< 0.001	66 (84.6)	0 (0.0)	< 0.001
High ACPA > 340 U/ml	16 (50.0)	0 (0.0)	< 0.001	25 (32.1)	0 (0.0)	< 0.001
DAS28-ESR at cut-off, mean (SD)	3.0 (1.1)	-	-	2.8 (1.0)	-	-
Remission-low activity, *n* (%)	20 (62.5)	-	-	51 (65.4)	-	-
Moderate-high activity, *n* (%)	12 (37.5)	-	-	27 (34.6)	.	-
Cumulative DAS28-ESR, mean (SD)	3.6 (0.5)	-	-	2.8 (0.6)	-	-
Remission-low activity, *n* (%)	16 (50.0)	-	-	55 (70.5)	-	-
Moderate-high activity, *n* (%)	16 (50.0)	-	-	23 (29.5)	-	-
Cumulative HAQ, median (IQR)	0.8 (0.6)	-	-	0.7 (0.6)	-	-
Synthetic DMARD, *n* (%)	30 (93.8)	0 (0.0)	< 0.001	71 (91.0)	0 (0.0)	< 0.001
Biologic DMARD, *n* (%)	11 (34.4)	0 (0.0)	< 0.001	31 (39.7)	0 (0.0)	< 0.001
Corticosteroids at cut-off, *n* (%)	5 (15.6)	0 (0.0)	0.020	14 (17.9)	0 (0.0)	< 0.001
*Collinsella aerofaciens* abundance, median (IQR)	0.005 (0.0009-0.01)	0.003 (0.0008–0.005)	0.035	0.007 (0.002–0.01)	0.002 (0.0005–0.008)	< 0.001
*Collinsella aerofaciens* abundance (OTU ≥ 0.010), *n* (%)	11 (34.4)	3 (9.4)	0.016	31 (39.7)	14 (18.7)	0.004

Abbreviations: RA: rheumatoid arthritis; ACPA: anti–citrullinated peptide antibody; RF: rheumatoid factor; SD: standard deviation; DAS28-ESR: 28-joint Disease Activity Score with erythrocyte sedimentation rate; CRP: C-reactive protein; DMARD: disease-modifying antirheumatic drug

Table 2 shows the differences between RA patients with and without the severe phenotype from the discovery and validation cohorts. As was to be expected, compared with patients with nonsevere disease, patients with severe RA in both cohorts had a higher average DAS28-ESR value, poorer physical functioning according to the HAQ, and high ACPA values (> 340 U/ml). Moreover, patients with severe RA in the discovery cohort had a greater abundance of *Collinsella*, more frequently had erosions, and more frequently took biologics. In contrast, there were no significant differences in disease duration between patients with severe and nonsevere RA within each cohort (discovery cohort: *p* = 0.358; validation cohort: *p* = 0.793).

**Table 2:** Characteristics of RA according to severity

	Discovery cohort			Validation cohort		
	Severe RA *N* = 16	Nonsevere RA *N* = 16	*p*-value	Severe RA *N* = 21	Nonsevere RA *N* = 57	*p*-value
*Epidemiological characteristics*						
Age, years, mean (SD)	60.2 (7.6)	55.7 (10.3)	0.178	57.4 (12.4)	54.1 (11.1)	0.260
Female sex, *n* (%)	12 (75.0)	12 (75.0)	1.000	15 (71.4)	49 (86.0)	0.138
Caucasian race, *n* (%)	16 (100.0)	16 (100.0)	1.000	21 (100.0)	55 (96.5)	0.358
BMI, kg/m^2^, mean (SD)	30.2 (6.2)	30.5 (5.5)	0.884	28.7 (5.1)	27.1 (4.3)	0.176
Smoking			0.060			0.001
Never, *n* (%)	4 (25.0)	9 (56.3)		2 (9.5)	32 (56.1)	
Ex-smoker, *n* (%)	8 (50.0)	2 (12.5)		9 (42.9)	11 (19.3)	
Active, *n* (%)	4 (25.0)	5 (31.3)		10 (47.6)	14 (24.6)	
*Comorbid conditions*						
Arterial hypertension, *n* (%)	4 (25.0)	6 (37.5)	0.694	6 (28.6)	13 (22.8)	0.599
Diabetes mellitus, *n* (%)	2 (12.5)	0 (0.0)	0.144	1 (4.8)	3 (5.3)	0.929
Dyslipidemia, *n* (%)	6 (37.5)	5 (31.3)	0.465	7 (33.3)	6 (10.5)	0.017
Obesity WHO, *n* (%)	9 (56.3)	7 (43.8)	0.669	7 (33.3)	16 (28.1)	0.148
*Clinical-laboratory characteristics*						
Duration of RA, months, mean (SD)	72.7 (14.7)	79.8 (18.2)	0.358	95.6 (37.6)	90.6 (26.2)	0.793
Diagnostic delay, months, mean (SD)	11.4 (8.5)	6.7 (4.2)	0.401	10.8 (9.2)	6.9 (8.9)	0.282
Erosions, *n* (%)	13 (81.3)	6 (37.5)	0.012	14 (66.7)	35 (61.4)	0.670
RF > 10 U/ml, *n* (%)	16 (100.0)	13 (81.3)	0.069	18 (85.7)	45 (78.9)	0.501
ACPA > 20 U/ml, *n* (%)	16 (100.0)	7 (43.8)	< 0.001	18 (85.7)	48 (84.2)	0.870
High ACPA > 340 U/ml	11 (68.8)	5 (31.1)	0.034	12 (57.1)	13 (22.8)	0.004
DAS28-ESR at cut-off, mean (SD)	3.2 (1.2)	2.8 (1.0)	0.330	3.4 (0.5)	2.6 (0.4)	< 0.001
Cumulative DAS28, mean (SD)	3.6 (0.6)	2.9 (0.7)	0.017	3.6 (0.9)	2.5 (0.8)	< 0.001
Cumulative HAQ, median (IQR)	1.1 (0.7)	0.9 (0.5)	0.080	1.0 (0.6)	0.6 (0.5)	0.006
Synthetic DMARD, *n* (%)	15 (93.8)	16 (100.0)	0.310	17 (81.0)	54 (94.7)	0.059
Biologic DMARD, *n* (%)	9 (56.3)	2 (12.5)	0.009	10 (47.6)	21 (36.8)	0.388
Corticosteroids at cut-off, *n* (%)	2 (12.5)	0 (0.0)	0.365	4 (19.0)	10 (17.4)	0.878
*Collinsella* abundance, median (IQR)	0.011 (0.001–0.026)	0.002 (0.0003–0.007)	0.021	0.011 (0.003–0.025)	0.006 (0.002–0.01)	0.142
*Collinsella aerofaciens* abundance (OTU ≥ 0.10), *n* (%)	9 (100.0)	8 (50.0)	0.016	10 (47.6)	21 (36.8)	0.388

Abbreviations: RA: rheumatoid arthritis; ACPA: anti–citrullinated peptide antibody; RF: rheumatoid factor; SD: standard deviation; DAS28-ESR: 28-joint Disease Activity Score with erythrocyte sedimentation rate; CRP: C-reactive protein; DMARD: disease-modifying antirheumatic drug

### Differentially methylated positions

A comparative analysis of genome-wide CpG methylation levels was performed in RA patients and controls, as well as in patients with severe and nonsevere disease. The comparison of patients and controls revealed that 52,430 CpG sites were DMPs, and the comparison of severe and nonsevere RA revealed that 83,326 CpG sites were DMPs (*p* < 0.05). Of these DMPs, 47 CpGs and 169 CpGs presented significant differences in absolute methylation values (|Δβ| ≥ 0.10) between patients and controls and between severe and nonsevere RA, respectively.

**Fig. 1:**
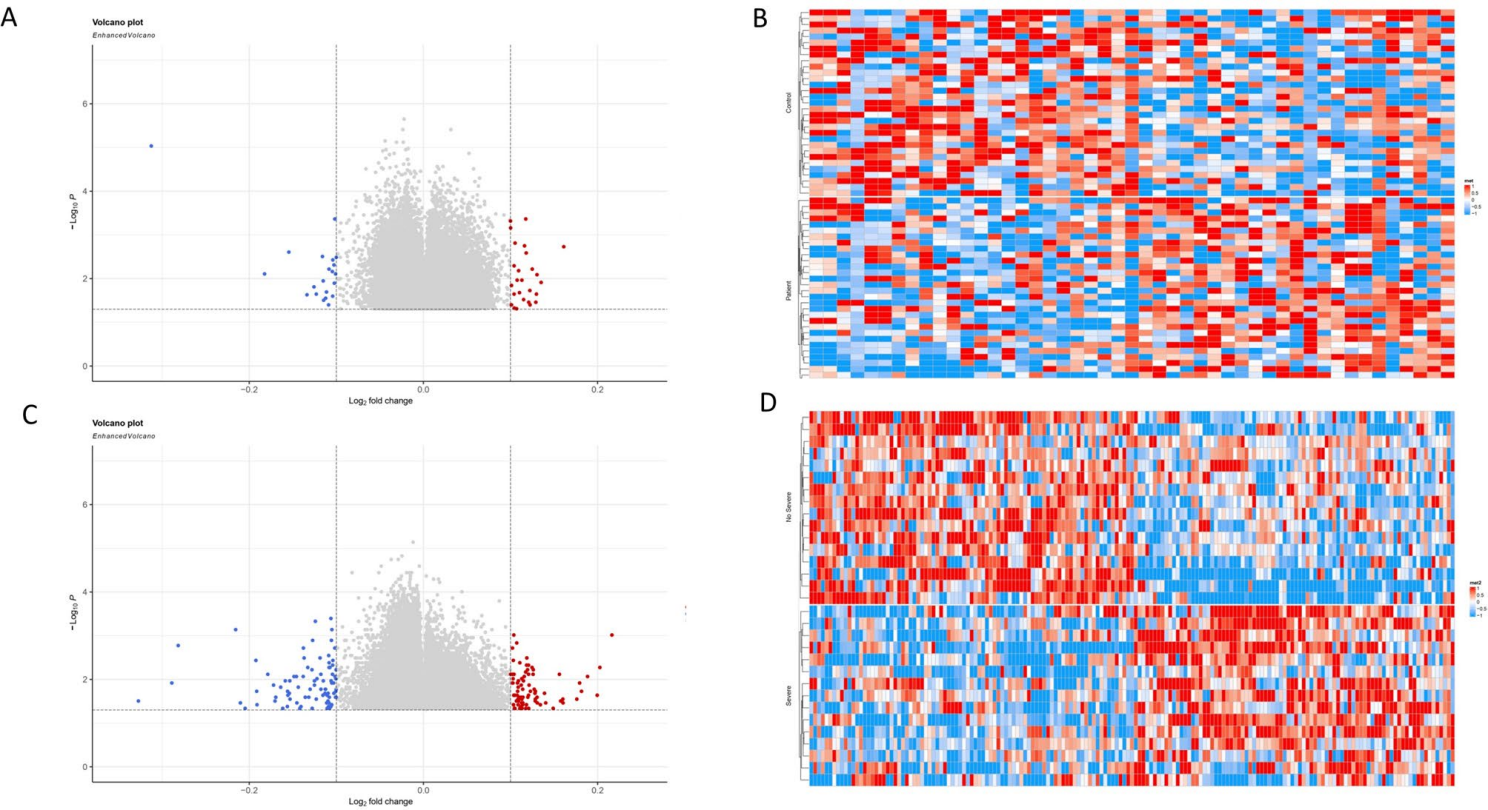
Differentially methylated positions (DMPs) associated with RA and RA severity. (**A**, **C**) Volcano plot of differentially methylated positions (DMPs) in patients versus controls (A) and severe RA versus nonsevere RA (C). The DMPs in the left and right quadrants (hypermethylated in red, hypomethylated in blue) are differentially methylated with a *p*-value ≤ 0.05 and|Δβ| ≥0.1. (**B**, **D**) Heatmap generated from the 47 DMPs obtained in the RA group compared to the control group (**B**) and 169 DMPs obtained in the severe RA group compared to the nonsevere RA group (**D**). The rows represent each DMP and the columns each participant (patients and controls). The colors represent the methylation levels: the redder the color, the more methylated the position, and the bluer the color, the less methylated the position

For better visualization of methylation status, volcano plots and a heatmap were created from the methylation values of each DMP obtained from the comparisons between patients and controls and between severe RA and nonsevere RA (*p* ≤ 0.05 and |Δβ| ≥ 0.1) (Fig. 1). The heatmap displayed the specific methylation value of each CpG site for each patient and clearly identified 2 distinct profiles in both analyses: patients versus controls and severe versus nonsevere cases, as shown in Fig. 1B and D, respectively.

**Fig. 2:**
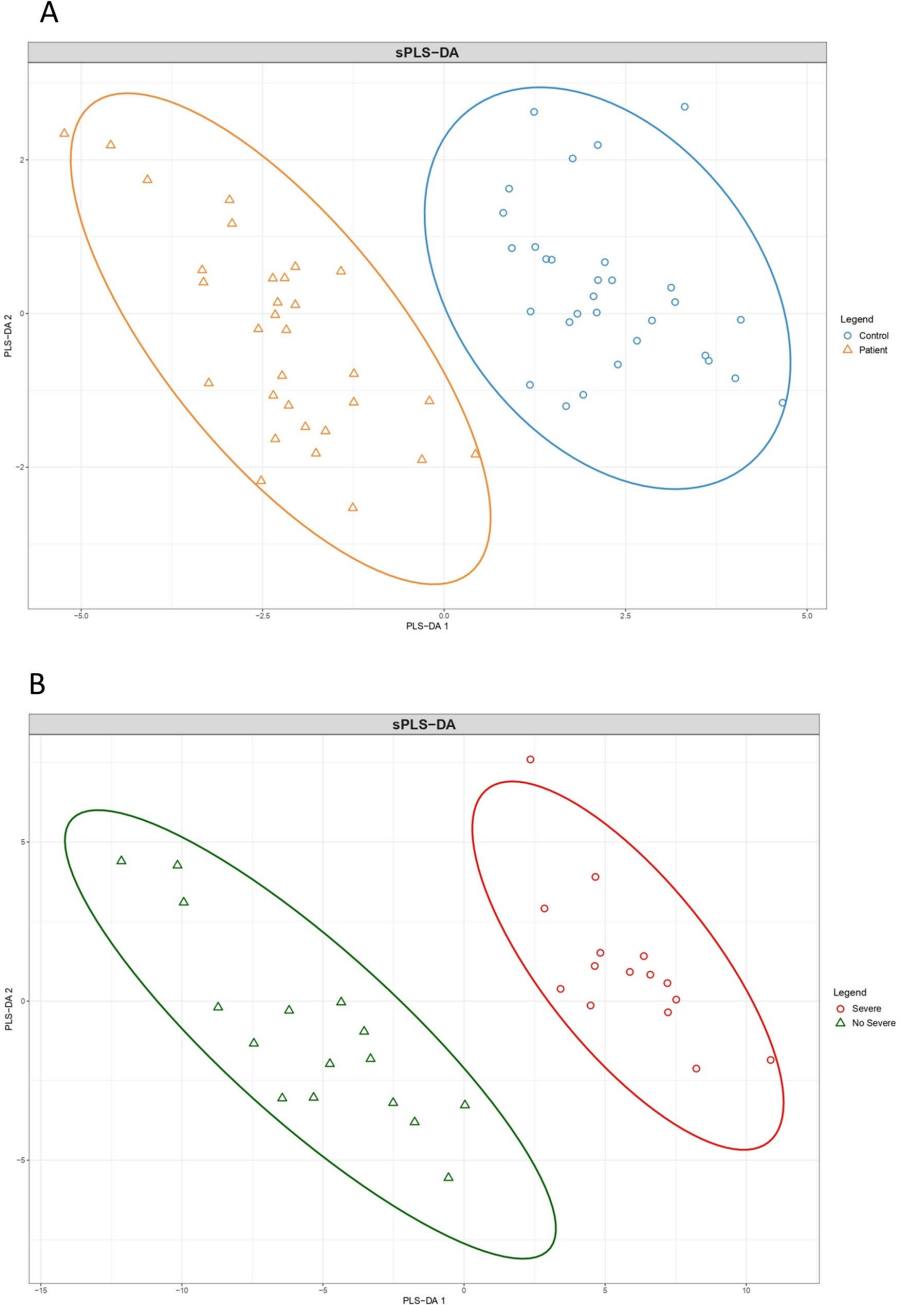
Principal component analysis for (**A**) patients (orange, cluster 1) and controls (blue, cluster 2), and (**B**) nonsevere RA (green, cluster 1) and severe RA (red, cluster 2)

The PCA analysis shown in Fig. 2 illustrates the global differences in CpG methylation patterns between the groups compared. As can be seen, the significant CpG sites identified have sufficient discriminatory capacity to form a distinct cluster of RA patients separate from the healthy controls (Fig. 2, A). Patients with severe RA also clustered separately from patients with nonsevere RA (Fig. 2, B). These loci were distributed, respectively, over 28 genes (12 hypermethylated genes, 16 hypomethylated genes) and 111 genes (57 hypermethylated genes, 54 hypomethylated genes). The top 25 DMPs (annotated in genes) associated with RA and RA severity are presented according to |Δβ| in Tables 3 and 4, respectively.

**Table 3:** Top 25 DMPs associated with RA (RA patients vs. healthy controls) in blood DNA

CpG	Relation to CpG	Gene	Relation to Gene	*p*-value	|Δβ|
cg22444562	OpenSea	*GPR1-AS*	Body	0.008	0.18
cg02484732	OpenSea	*COL21A1*	5’UTR	0.023	0.13
cg26116556	OpenSea	*PLEKHG1*	5’UTR	0.015	0.13
**cg21950155**	S_Shore	***PRHOXNB***	Body	**0.003**	**0.12**
cg13077366	OpenSea	*BRUNOL4*	Body	0.011	0.11
cg23658987	N_Shore	*TNN*	Body	0.031	0.11
cg09972436	OpenSea	*LCE3C*	TSS1500	0.028	0.11
cg26370237	OpenSea	*HSF5*	Body	0.020	0.11
cg26095158	OpenSea	*LYPLAL1-AS1*	Body	0.040	0.11
cg17894854	OpenSea	*EPHA5*	Body	0.006	0.11
**cg08161306**	OpenSea	***TBC1D22A***	TSS1500;5’UTR; Body	**0.003**	**0.10**
cg18389601	OpenSea	*NARFL*	Body;5’UTR	0.000	-0.10
**cg05510714**	OpenSea	***KYNU***	Body	**0.005**	**-0.10**
cg26408224	OpenSea	*MSI2*	Body	0.002	-0.11
cg26954695	OpenSea	*MZF1;MGC2752;LOC100131691*	TSS1500;Body	0.049	-0.11
cg11063088	OpenSea	*DLG5*	Body	0.021	-0.11
cg12011299	OpenSea	*ADH4*	TSS200	0.011	-0.11
cg16972032	OpenSea	*SERINC5*	Body	0.002	-0.12
cg22405820	OpenSea	*C8orf37-AS1*	Body	0.000	-0.12
cg04954225	OpenSea	*RGS6*	Body	0.003	-0.12
cg07469075	OpenSea	*PAMR1*	TSS1500;5’UTR	0.019	-0.12
cg26999053	OpenSea	*MYH10*	Body	0.040	-0.12
cg03370588	OpenSea	*MYO5A*	TSS200	0.023	-0.13
cg27562174	OpenSea	*ADH4*	5’UTR;1stExon; Body	0.008	-0.13
cg03126799	OpenSea	*LOXL2*	Body	0.012	-0.14

In bold: CpG site selected for the pyrosequencing validation study

**Table 4:** Top 25 DMPs associated with RA severity (severe RA vs. nonsevere RA) in blood DNA

CpG	Relation to GpC	Gene	Relation to Gene	*p*-value	|Δβ|
**cg05073382**	**N_Shore**	***MYOM2***	**Body**	**0.001**	**-0.22**
cg17480035	S_Shore	*HLA-DRB1*	Body	0.034	0.29
**cg15971518**	**OpenSea**	***PRG2***	**TSS1500**	**0.001**	**0.28**
cg01578633	OpenSea	*PRG2*	TSS1500	0.001	0.22
cg00481382	OpenSea	*NEDD1*	5’UTR; Body	0.009	0.19
cg00322003	OpenSea	*WNK1*	Body	0.019	0.18
cg08586441	OpenSea	*TEC*	Body	0.012	0.18
cg19978674	OpenSea	*HOPX*	TSS1500, 5’UTR	0.034	0.16
cg23174406	OpenSea	*NEDD1*	Body;5’UTR	0.028	0.16
cg11787167	OpenSea	*NPAS3*	TSS1500	0.031	0.16
**cg19052272**	**OpenSea**	***ALLC***	**TSS1500**	**0.009**	**-0.15**
cg11079896	OpenSea	*RHOJ*	5’UTR;1stExon	0.023	-0.16
cg23066280	OpenSea	*PTPRN2*	Body	0.042	-0.16
cg21787089	OpenSea	*LTF*	Body	0.011	-0.16
cg07189587	OpenSea	*RHOJ*	5’UTR;1stExon	0.046	-0.16
cg24587835	OpenSea	*LOC339166*	TSS1500	0.015	-0.16
cg07157030	OpenSea	*RHOJ*	5’UTR;1stExon	0.031	-0.17
cg26805839	OpenSea	*SLC1A1*	Body	0.013	-0.17
cg16474696	OpenSea	*MRI1*	TSS1500	0.008	-0.18
cg01427108	OpenSea	*LTF*	Body	0.038	-0.19
cg25755428	OpenSea	*MRI1*	TSS1500	0.004	-0.19
cg00670575	OpenSea	*SHANK2*	5’UTR	0.034	-0.21
cg25721006	OpenSea	*LHPP*	TSS1500	0.002	-0.28
cg05872034	OpenSea	*NPHP4*	Body	0.012	-0.29
cg10576280	N_Shore	*PLEKHA1*	TSS1500	0.031	-0.33

In bold: CpG site selected for the pyrosequencing validation study

Potential biomarkers for validation were selected from the top 25 DMPs, as follows: cg21950155, cg08161306, cg05510714, cg05073382, cg15971518, and cg19052272. In addition to having a |Δβ| ≥ 0.10 and being annotated in genes, these biomarkers had a *p*-value < 0.01 and formed part of the DMRs (Table S2).

Moreover, overlap and dispersion of data between groups was taken into consideration (data not shown). Although cg15971518 was not among the DMRs, it was added to the group of CpGs, since another relevant DMP (top 25) was identified in the same gene and fulfilled the selection criteria.

### Enrichment analysis (DMPS annotated in genes)

To perform the functional analysis, we selected a total of 1137 genes associated with the DMPs in patients and controls and 1229 genes associated with DMPs in severe RA and nonsevere RA (|Δβ| >0.05 and *p <* 0.05). The GO analysis showed that DMPs identified between patients and controls were mostly enriched in four molecular functions (Figure S1, A), the most important of which were regulation and activation of GTPase, molecular functions that, in the molecular analysis, were associated with genes from the TBC1D family, including the gene *TBC1D22A*, whose DMP (cg08161306) was previously proposed as a potential biomarker of RA (see above). Figure S1 (B) shows the results of the GO analysis for the DMPs between patients with severe and nonsevere RA. The KEGG pathway analysis revealed the 10 most relevant pathways according to the number of genes involved in relation to RA and RA severity (Figure S2). The pathway related to purine metabolism is particularly interesting owing to the presence of the *ALLC* gene, which was previously selected (cg19052272) for validation and proposed as a possible biomarker of severity of RA. Furthermore, the group of genes associated with DMPs were distributed along all the pathways of purine metabolism, indicating modulation in almost all alternative pathways (Figure S3).

### Validation of candidate cpg sites

In the validation study of the DMPs selected in the HM850K array (cg21950155, cg08161306, cg05510714, cg05073382, cg15971518, and cg19052272), the only CpG sites that were technically confirmed by pyrosequencing were those associated with RA and severity of RA shown in Tables 3 and 4. These technically verified CpG sites were subsequently transferred to an independent validation cohort (Table 3).

In the discovery cohort, the methylation levels of the pyrosequenced CpGs were higher in patients overall and in patients with more severe RA than in the controls and patients with less severe disease, respectively, except for cg19052272 and 5-Down-cg19052272 (Tables 3 and 4). In the validation cohort, methylation levels were higher for 19-Down-cg08161306 in patients, thus replicating the results obtained in the discovery cohort. Methylation levels of 7-Down-cg08161306, an adjacent CpG site, were also higher in patients with severe RA (Table 5).

**Table 5:** Mean methylation levels obtained by pyrosequencing of selected CpGs for the study groups

Gene	ID	Discovery Cohort	Validation Cohort
		RA Patients vs. Controls	RA Patients vs. Controls
*PRHOXNB*	cg21950155	60.45 ± 16.11 vs. 47.06 ± 14.123, *p* = 0.001	53.78 ± 15.68 vs. 53.46 ± 14.85, *p* > 0.05
*TBC1D22A*	cg08161306	75.08 ± 13.92 vs. 63.96 ± 13.54, *p* = 0.002	73.18 ± 14.83 vs. 71.92 ± 15.75, *p* > 0.05
	7-Down-cg08161306	86.35 ± 5.43v s. 79.46 ± 6.65, *p* < 0.0001	84.58 ± 6.80 vs. 83.32 ± 7.79, *p* > 0.05
	19-Down-cg08161306	81.23 ± 4.81 vs. 74.48 ± 6.04, *p* < 0.0001	80.10 ± 5.70 vs. 77.96 ± 6.41, *p* = 0.03
		Severe RA vs. Nonsevere RA	Severe RA vs. Nonsevere RA
*PRG2*	cg15971518	37.09 ± 14.17 vs. 18.1762 ± 8.86, *p* < 0.0001	28.14 ± 14.68 vs. 29.57 ± 14.86, *p* > 0.05
*ALLC*	5-Down-cg19052272	55.04 ± 12.21 vs. 65.63 ± 6.10, *p* = 0.005	59.63 ± 16.13 vs. 61.98 ± 10.72, *p* > 0.05
	cg19052272	75.73 ± 13.10 vs. 87.70 ± 5.43, *p* = 0.003	79.05 ± 18.75 vs. 83.80 ± 11.24, *p* > 0.05
*TBC1D22A*	7-Down-cg08161306	85.60 ± 5.32 vs. 87.10 ± 5.60, *p* > 0.05	87.32 ± 7.06 vs. 83.57 ± 6.47, *p* = 0.031

Abbreviation; RA: rheumatoid arthritis

### Association between dmps and RA and severity of RA

All the CpG sites validated using pyrosequencing, which were characterized by significant differences in their means, were associated with RA or severity of RA in a logistic regression analysis (Table 6). The increase in the methylation levels of cg21950155, cg08161306, 7 and 19 Down-cg08161306, and cg15971518 was associated with a greater risk and severity of RA. However, the increase in the methylation levels of cg19052272 and 5-Down-cg19052272 reduced the risk of severity of RA (Table 6).

**Table 6:** Association between DMPs and RA and severity of RA: logistic regression

Gene	ID	Discovery Cohort	Validation Cohort
		Patients vs. Controls	Patients vs. Controls
*PRHOXNB*	cg21950155	R^2^ = 0.163 OR: 1.059, 95% CI (1.02–1.09), *p* = 0.002*	*p* > 0.05
*TBC1D22A*	cg08161306	R^2^ = 0.143 OR: 1.062, 95% CI (1.01–1.10), *p* = 0.004*	*p* > 0.05
	7-Down-cg08161306	R^2^ = 0.247 OR: 1.210, 95% CI (1.08–1.34), *p* < 0.0001*	*p* > 0.05
	19-Down-cg08161306	R^2^ = 0.280 OR: 1.260, 95% CI (1.113–1.432), *p* < 0.0001*	R^2^ = 0.030 OR:1.060, 95% CI (1.005–1.18), *p* = 0.032*
		Severe RA vs. Nonsevere RA	Severe RA vs. Nonsevere RA
*PRG2*	cg15971518	R^2^ = 0.385 OR: 1.130, 95% CI (1.04–1.22), *p* = 0.002*	*p* > 0.05
*ALLC*	5-Down-cg19052272	R^2^ = 0.233 OR: 0.89, 95% CI (0.82–0.97), *p* = 0.01*	*p* > 0.05
	cg19052272	R^2^ = 0.263 OR: 0.89, 95% CI (0.81–0.97), *p* = 0.01*	*p* > 0.05
*TBC1D22A*	7-Down-cg08161306	*p* > 0.05	R^2^ = 0.060 OR:1.089, 95% CI (1.006–1.178), *p* = 0.034*

The table shows the results of two logistic regression models. Model 1: dependent variable: Patients (1) vs. Controls (0). Model 2: dependent variable: Severe RA (1) vs. Nonsevere RA (0). *p < 0.05 with age and sex also included in the model

No significant differences were observed between severe and nonsevere RA for cg15971518 and cg19052272, although the methylation values for these CpGs were positively correlated with inflammation-related variables such as DAS28-ESR (*r* = − 0.276 [*p* = 0.010] and *r* = 0.256 [*p* = 0.029] for cg15971518 and cg19052272, respectively). Furthermore, cg15971518 was associated with DAS28-ESR in a linear regression model adjusted for sex and age (β = − 0.257, *p* = 0.028, R^2^ = 0.050). Similarly, no significant differences in methylation were recorded for cg21950155 between patients and controls, although this CpG was associated with RA-related variables such as physical function according to the HAQ (*r* = 0.275, *p* = 0.017).

### Diagnostic potential of DMPS validated by pyrosequencing

**Fig. 3:**
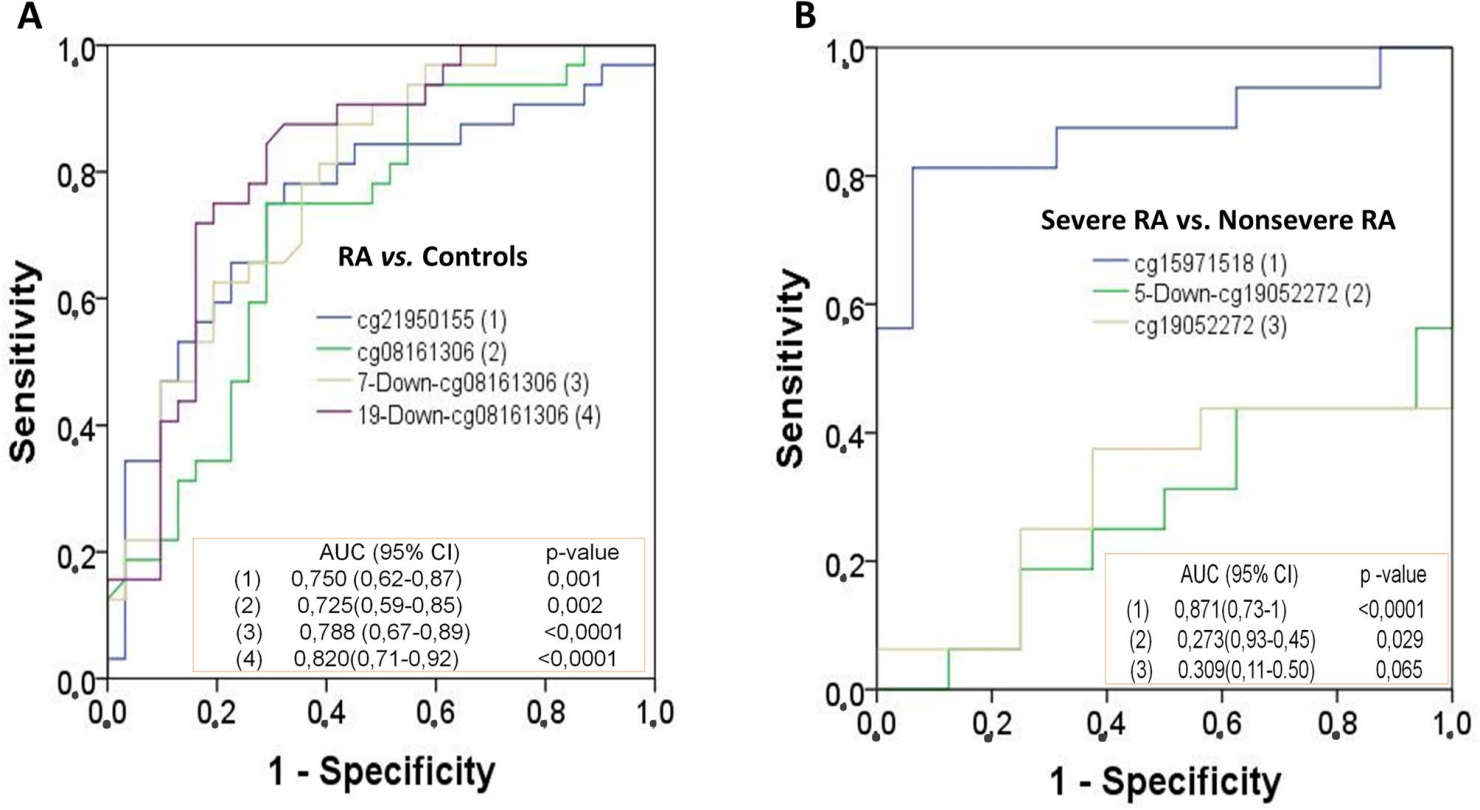
Diagnostic potential of methylation levels of validated CpGs in the discovery cohort for differentiating between patients with RA and controls (**A**) and severe RA and nonsevere RA (**B**)

To evaluate the clinical performance of methylation in the diagnosis and severity of RA, we constructed ROC curves and calculated the AUC. Figure 3 shows the discriminative ability of methylation levels for the CpGs validated in the discovery cohort. The AUC results showed that methylation of 19-Down-cg08161306 and cg15971518 was more able to distinguish between RA patients and controls and between severe and nonsevere RA, respectively (Fig. 3). The combination of the four DMPs with discriminative power in patients compared with controls increased the AUC (0.877 [0.794–0.960], *p* < 0.0001). Similarly, the diagnostic power resulting from the combination of the three DMPs in severely ill patients compared with nonseverely ill patients was stronger with respect to each DMP individually (0.891 [0.768-1], *p* < 0.0001).

In the validation cohort, 19-Down-cg08161306 was the only CpG that maintained its diagnostic ability to distinguish between RA patients and healthy controls, albeit with a reduced AUC (0.609 [0.520–0.698]; *p* = 0.019).

## Discussion

Epigenetic modifications can affect the development and progression of RA; therefore, it is crucial to identify epigenetic biomarkers in affected patients [25]. The present study compared methylation patterns between patients with RA and healthy controls and between patients with and without severity factors. Such an analysis makes it possible to identify potential biomarkers of methylation associated with susceptibility to and severity of RA.

By analyzing 485,000 CpG sites throughout the genome, we identified a specific methylation profile associated with RA and severity of RA.

By analyzing 850,000 CpG sites across the genome using the Illumina EPIC array, we identified a specific methylation profile associated with RA and the severity of RA. We observed significant changes at 47 CpG sites when comparing patients with RA and controls and at 169 CpG sites when comparing severe and nonsevere cases. Some of these CpGs were located in 28 and 111 genes respectively, and could constitute an epigenetic signature for risk and severity of RA. Moreover, in an independent cohort, we verified that the 19-Down-cg08161306 site in *TBC1D22A* was hypermethylated in patients with RA. Interestingly, most of the genes we found to be differentially methylated are known to play a functional role in the inflammatory response.

Several studies searching for epigenetic biomarkers have been performed in twins and relatives of RA patients, although few have been based on whole genome analysis [26–31]. One such study confirmed the role of the *RGS1* gene in patients with RA [26]. This gene has been widely studied in tumors but not in RA patients. The same authors suggest that inhibiting expression of *RGS1* could inhibit the inflammatory response and angiogenesis. In line with these results, we identified methylation abnormalities in *RGS6*, another gene from the same family, pointing to its possible involvement in RA and opening new lines of research. *RGS6* plays a protective role in acute inflammation models, not only by acting on the early immune response, but also on regeneration [32]. Methylation abnormalities were also found in the DNA of genes from the solute carrier family (SLC) in patients with RA, for example, *SLC1A1* in our study and *SLC2A12* elsewhere [27]. Furthermore, while some studies have identified DMP genes associated with RA [28–30], very few have sought to identify epigenetic biomarkers associated with the severity of RA [31].

In the present study, pyrosequencing-based technical validation of the CpG sites selected confirmed several DMPs to be associated both with the risk of RA (cg21950155 [gene *PRHOXNB*] and cg08161306 [gene *TBC1D22A*]) and with the severity of RA (cg15971518 [gene *PRG2*] and cg19052272 [gene *ALLC*]). Interestingly, the genes we validated have GO annotations that may be indirectly related to inflammation or autoimmunity. For example, the PRHOXNB gene (cg21950155) encodes a putative enzyme involved in purine metabolism and urate degradation to (S)-allantoin, a pathway in which altered levels have been reported in patients with RA [33].

Another relevant finding of our study was, for the first time, an association between methylation in the *TBC1D22A* gene and RA. This finding was confirmed both technically and experimentally and, according to the area under the ROC curve, it may act as a diagnostic marker. Although abnormalities in this gene are associated with severe neurological diseases, some of its variants have been involved in the production of cytokines in response to pathogens [34]. *TBC1D22A* is a protein-coding gene located at chromosome 22 that probably participates in activation of Rab-GTPase activity and intracellular protein transport [35]. Rab-GTPase proteins are involved in innate and adaptive immunity via PI3 kinase [36–38] and induce formation of neutrophil extracellular traps (NETs). Specifically, Rab 5a could aggravate RA by forming NETS that induce inflammation of macrophages and secretion of inflammatory cytokines [39].

As for the gene *PRG2*, which encodes the proteoglycan 2 protein, methylation levels at the cg15971518 site were superior in patients with severe RA. While *PRG2* had not been previously related to RA, it had been associated with hypereosinophilic syndrome [40]. Hypereosinophilia can occur in cases of severe RA and chronic disease, especially in patients with extra-articular manifestations and high RF titers [41]. Methylation levels in *PGR2* also differentiated between patients with severe and nonsevere disease (area under the ROC curve, 0.87).

For its part, the *ALLC* gene, which was also associated with modifications in methylation in RA patients, codes for allantoicase, an enzyme that participates in purine metabolism and uric acid degradation. Although the activity of allantoicase was lost during the evolution of vertebrates, our study associated methylation of *ALLC* with the severity of RA. We found that the increased methylation in 2 CpGs in the gene promoter (5-Down-cg19052272 and cg19052272), which, in theory, reduces gene expression, was associated with a lower risk of severe disease. The literature supports the involvement of this gene—and others—in purine metabolism [42].

Finally, although they replicated the results for the array, the DMPs cg21950155, cg15971518, cg19052272, and 5-Down-cg19052272 were not associated with RA or with severity of RA in the validation cohort, possibly because of the presence of more extreme phenotypes in the discovery cohort. During the discovery phase, patients were grouped into 2 extreme phenotypes based on various parameters, such as inflammatory activity according to the DAS28-ESR (low or moderate-high activity), presence or absence of RF and ACPA, and abundance of *Collinsella aerofaciens* (high or not). In contrast, the validation cohort was formed based only on the DAS28-ESR (low or moderate-high activity). This characteristic could be a limitation of the study.

Our study is subject to a series of limitations. Despite having both a discovery and a validation cohort, stratification into extreme phenotypes may not account for all patients with RA, especially those with intermediate phenotypes. However, this strategy enabled us to detect more marked differences in methylation patterns and to increase the statistical power of the study. Another limitation is that the participation of the intestinal microbiota as a potential severity factor was only assessed with respect to the abundance of *Collinsella aerofaciens*. However, the selection of this bacterial species was based on previous evidence pointing to its relevance in the pathogenesis of RA by our research group and by other studies [3, 43]. Moreover, the integration of DNA methylation data with detailed clinical characteristics and the abundance of *Collinsella aerofaciens* provides a more holistic perspective of the disease, since it helps us to understand how the microbiota and epigenetic modifications interact to affect RA. Also, due to the moderate sample size and subtle methylation differences observed, significant CpG islands identified by the Wilcoxon test did not remain significant after multiple testing correction. Larger studies or stronger methylation effects are required to confirm these findings. Finally, the observational and cross-sectional design also constitutes a major limitation because it may not reflect dynamic changes in the patient’s situation or in methylation patterns over time. This limitation is mitigated in part by the fact that the patients are from a prospective and dynamic cohort of early RA patients that made it possible to use standardized covariates of inflammatory activity and physical function in the disease throughout follow-up, directly from inclusion in the cohort. It is also mitigated by the exhaustive collection of epidemiological, clinical, laboratory, and treatment-related data.

## Conclusions

In conclusion, this study identified DMPs in two gene clusters consisting of 28 and 111 genes. These could constitute an epigenetic signature for predicting, respectively, the susceptibility to and severity of RA. This epigenetic signature involves genes such as *TBC1D22A*, *PRHOXNB*, *ALLC*, and *PRG2* and associates them with both the risk and severity of RA. Pyrosequencing-confirmed hypermethylation of *TBC1D22A* in RA patients revealed a novel association between methylation levels in the gene and the risk of developing disease. Moreover, methylation in *TBC1D22A* and other members of its family suggests an effect on the activity of Rab-GTPase, which is relevant to both innate and adaptive immunity. The study integrated DNA methylation data, clinical characteristics, and intestinal microbiota to provide a multidimensional and holistic vision of RA. These findings highlight the potential of epigenetic biomarkers in the management of RA and support future research since more studies are needed to explore the functionality of the genes identified and their role in the pathogenesis of RA. Finally, validation in larger and more diverse cohorts is needed to confirm these findings and their applicability in various populations.

## Supplementary Information

Below is the link to the electronic supplementary material.

Supplementary Material 1

## Data Availability

No datasets were generated or analysed during the current study.
