# Supplementary material for: Analysis of epigenetic biomarkers for diagnosis and assessment of severity in rheumatoid arthritis: a cross-sectional study
Source: Arthritis Res Ther. 2025 Aug 8;27:165. doi: 10.1186/s13075-025-03628-2 (PMC12333266; doi:10.1186/s13075-025-03628-2)
Supplement: Supplementary file 1 — Supplementary Material 1 [file 13075_2025_3628_MOESM1_ESM.pdf]

Table S1: Sequence of the primers used for pyrosequencing validation of selected CpGs.

CpG	Forward primer	Reverse primer	Sequencing primer
cg21950155	ATGTGTTTATTGGTTTGTAGTAAGG	**ATAACACCTACTTACCCAATATCC**	TGGTTTGTAGTAAGGATTT
cg08161306	GTGTTTGGTTTTGGGAGTATAAGATGA	**AACCCCAACCACCTCAATTCTAA**	TTGAGTAGTTTTTTTTTTTGAGGA
cg15971518	TGATGGAAGATGGGTTTAGGAGTTA	**CTACATCCAACCACTTACCATACATAACA**	GAGGATTTTTAAGTATAGGAAA
cg19052272	**AGTTAGTAGGTGATTTGGAAGTATATTAGA**	TCCCCCCCACAACAATACAATACC	CCTCCAATCAATCACAAAACTC
cg05073382	**TGGGTTAAATTATTATTATGTTGTGTAGGA**	ACTTCAATTTATCCTCCTCACT	ACACTCACAAAAACCC
cg05510714	TGTGTTTGGGAAATATAGTTGTAAAT	**CTATCTATCTCTTTTTAAACCCAAAACC**	TTATTTTTTTAGATGTATAATGATT

In bold: Biotinylated primer

Table S2: DNA differentially methylated regions (DMRs) in RA *vs.* Controls and RA severe *vs* RA nonsevere for the selected biomarkers**.**

**DMR**	**Relevant CpGs of the DMR**	**Gene**	**Region type**	**Direction of association (NES)**	***p*-value**
chr13:28554427-28555065	cg13156931<br>cg15332217<br>cg02645340<br>**cg21950155**<br>cg09781827<br>cg25591794<br>cg07911080	*PRHOXNB*	CGI	Hypermethylated (2,212)	7,09E-06*
chr13:28552610-28558413	cg13156931<br>cg15332217<br>cg02645340<br>**cg21950155**<br>cg09781827<br>cg25591794<br>cg07911080	*PRHOXNB*	Body	Hypermethylated (2,096)	0,0001*
chr22:47157914-47187251	cg03203801<br>cg16053386<br>cg01772186<br>cg02321845<br>cg25916883<br>**cg08161306**<br>cg15596311<br>cg08937831<br>cg19578790<br>cg17063127<br>cg22688098<br>cg14311282<br>cg07869308<br>cg21143561<br>cg17501352<br>cg03061223<br>cg15100262<br>cg17150328<br>cg02781602	*TBC1D22A*	Promoter	Hypermethylated (2,323)	4,58E-06*
chr22:47158756-47567620	cg16462450<br>cg03203801<br>cg16053386<br>cg26451411<br>cg11425737<br>cg16554928<br>cg02926356<br>cg01772186<br>cg02321845<br>cg11223753<br>cg16817594<br>cg25916883<br>cg25109235<br>cg18049984<br>cg26699747<br>cg07567533<br>**cg08161306**<br>cg02478836	*TBC1D22A*	Body	Hypermethylated (1,504)	0,001*

	cg15596311<br>cg05821487<br>cg00293439<br>cg08592682<br>cg21192034<br>cg09547053<br>cg04356590<br>cg19578790<br>cg01527394<br>cg02951230<br>cg06321741<br>cg17063127<br>cg06332964<br>cg22688098<br>cg09960067<br>cg25742191<br>cg20812050<br>cg14311282<br>cg05496900<br>cg27617060<br>cg00159552<br>cg17145402<br>cg05398922<br>cg23705082<br>cg06236445<br>cg21143561<br>cg21030800<br>cg02895051<br>cg26538513<br>cg22367136<br>cg20919820<br>cg08381586<br>cg02492808<br>cg25364613<br>cg23136177<br>cg15536864<br>cg13874050<br>cg13333922<br>cg03061223<br>cg15100262<br>cg17150328<br>cg07397612<br>cg21621464<br>cg07607066<br>cg12998731<br>cg16138453				
chr2:143642986-143792380	**cg05510714**<br>cg05976716<br>cg12604353<br>cg04112100<br>cg09881932<br>cg03614377<br>cg11337520<br>cg03184584<br>cg21039462<br>cg05738507<br>cg14108150<br>cg27139090	*KYNU*	Body	Hypomethylated (-1,684)	0,009

chr8:1999014-2092745	cg02133140<br>cg09584650<br>cg01295646<br>cg07248162<br>cg21816685<br>cg11424828<br>cg21847720<br>cg02618355<br>cg07503203<br>cg06054159<br>cg06445972<br>cg11541409<br>cg12401644<br>cg20188974<br>cg06440275<br>cg06216883<br>cg11040181<br>cg04146801<br>cg01233141<br>cg00874558<br>cg04100307<br>cg25478112<br>**cg05073382**<br>cg22728991<br>cg21899743<br>cg08462127<br>cg22740895<br>cg08496165	*MYOM2*	Body	Hypomethylated (-1,429)	0,016
chr2:3704363-3718215	cg25251562<br>**cg19052272**<br>cg00999904<br>cg19825600<br>cg10645314<br>cg06156640	*ALLC*	Promoter	Hypomethylated (-1,870)	0,003

Normalized Enrichment Score (NES), CpG Islands (CGI). Promoter includes TSS1500, TSS200, 5' untranslated region [UTR], 1stExon. * adjusted *p*-value <0.05

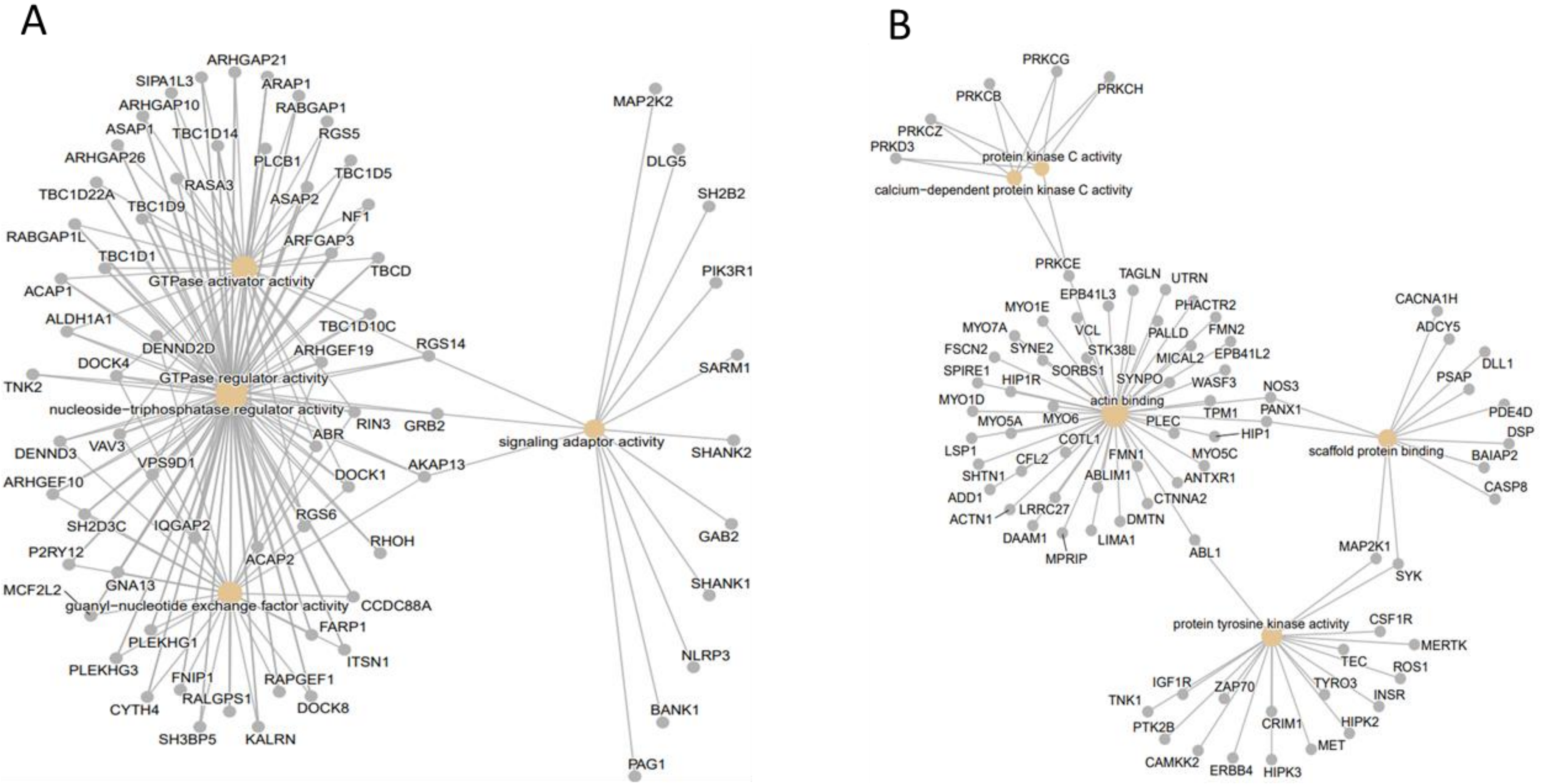

Figure S1: GO analysis for genes associated with DMPs between patients and controls (A) and severe RA vs nonsevere patients (B)

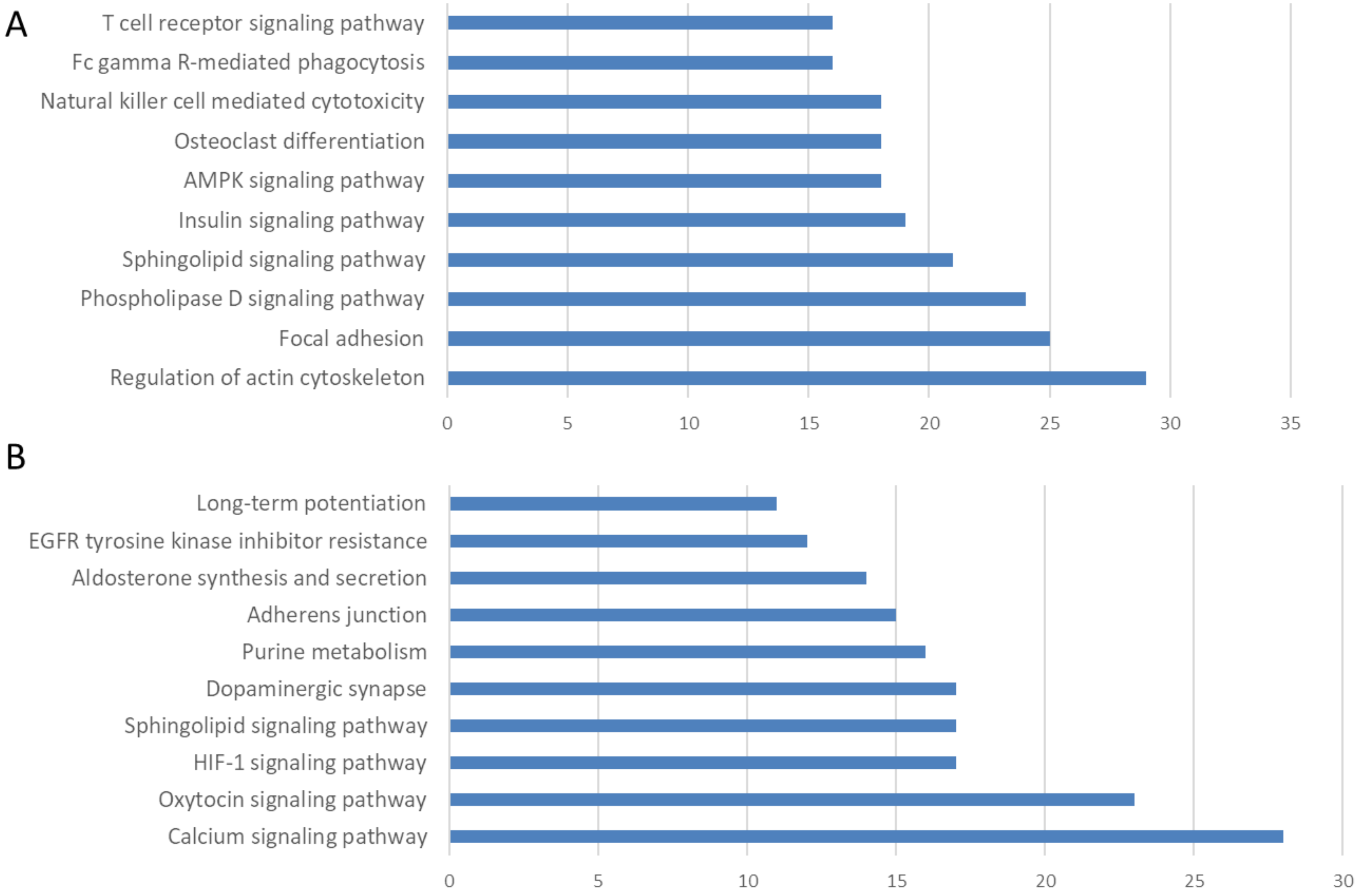

Figure S2: KEGG pathways most enriched (top 10) according to genes with DMPs between patients and controls (A) and severe and nonsevere RA (B). X-axis: gene frequency.

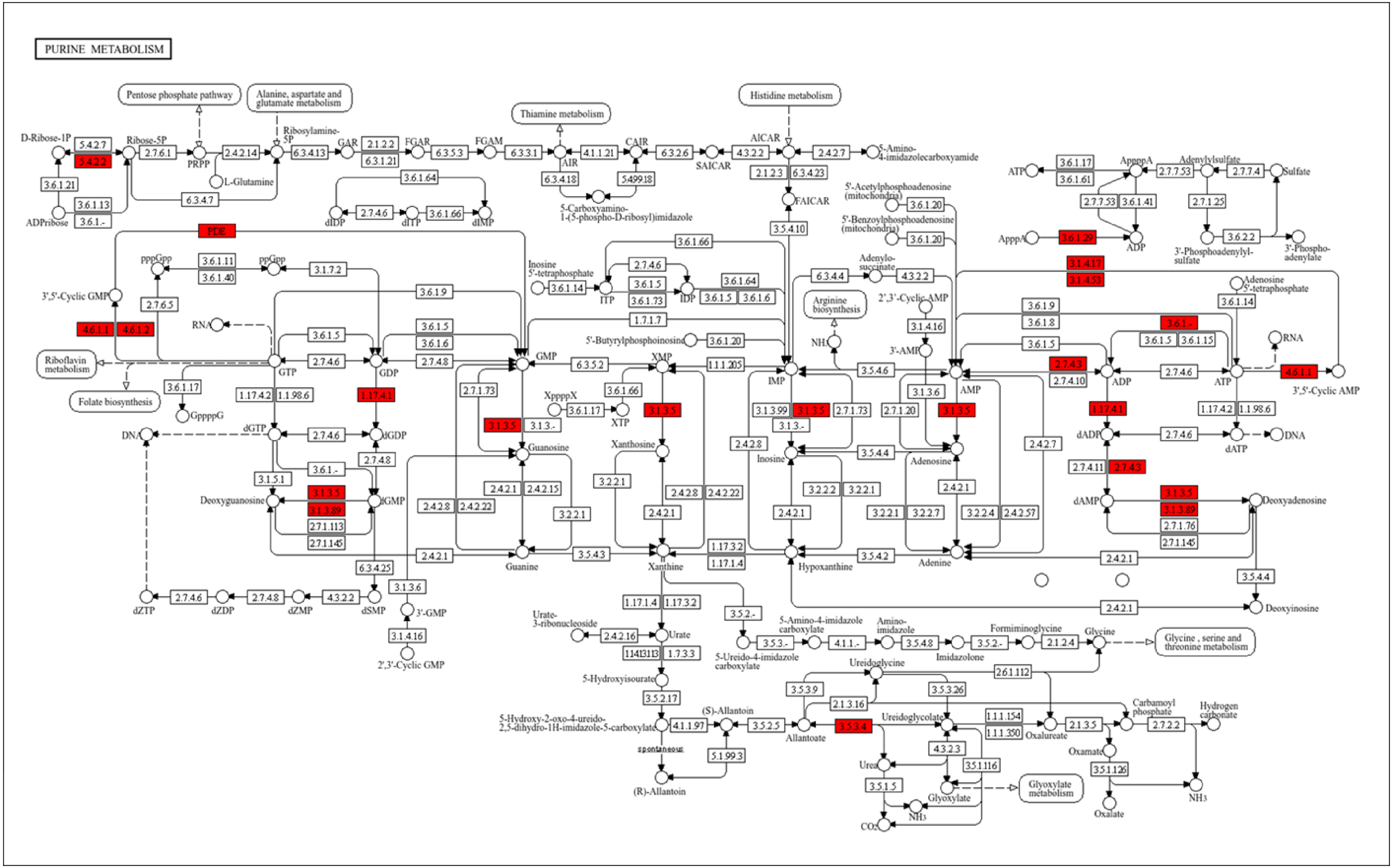

Figure S3: Purine Metabolism. Gene products of genes associated with DMPs have been marked in red.
